# Dendritic Spines as Tunable Regulators of Synaptic Signals

**DOI:** 10.3389/fpsyt.2016.00101

**Published:** 2016-06-09

**Authors:** Jan Tønnesen, U. Valentin Nägerl

**Affiliations:** ^1^Interdisciplinary Institute for Neuroscience, University of Bordeaux, Bordeaux, France; ^2^CNRS UMR 5297, Bordeaux, France

**Keywords:** synapses, synaptic plasticity, hippocampus, super-resolution fluorescence microscopy, dendritic spines

## Abstract

Neurons are perpetually receiving vast amounts of information in the form of synaptic input from surrounding cells. The majority of input occurs at thousands of dendritic spines, which mediate excitatory synaptic transmission in the brain, and is integrated by the dendritic and somatic compartments of the postsynaptic neuron. The functional role of dendritic spines in shaping biochemical and electrical signals transmitted via synapses has long been intensely studied. Yet, many basic questions remain unanswered, in particular regarding the impact of their nanoscale morphology on electrical signals. Here, we review our current understanding of the structure and function relationship of dendritic spines, focusing on the controversy of electrical compartmentalization and the potential role of spine structural changes in synaptic plasticity.

## Introduction

Dendritic spines harbor glutamatergic synapses and mediate the vast majority of excitatory synaptic transmission in the mammalian brain. They represent fundamental computational units of information processing that underlie sensory perception, emotions, and motor behavior. Spine structural and functional plasticity is an important substrate of learning and memory (1), while spine dysfunction is linked to neuropsychiatric and neurodegenerative disorders of the brain, including autism (2) and Alzheimer’s disease (3).

Ever since the discovery of dendritic spines by Ramon y Cajal more than a century ago, progress in understanding their anatomy and physiology has strongly depended on the development of new techniques to experimentally probe them (4).

Using the latest Golgi staining and light microscopy techniques of his days, Cajal hypothesized that spines harbor synapses and receive signals from other neurons (5). Still, it was not until 1959 that definitive proof for this idea was provided by the first electron microscopic (EM) images of synapse ultrastructure, revealing the presynaptic specialization, synaptic cleft, and postsynaptic density (PSD) (6).

Long before direct visualization of spine plasticity in live tissue became possible, EM provided the first hints of their dynamic nature, indicating that they change shape and size in response to repetitive synaptic stimulation (7). In parallel, theoretical studies formulated the idea that spines might compartmentalize biochemical and electrical signals, and thereby shape the functional properties of synapses (8–10).

The development of two-photon microscopy (11) opened up manifold opportunities to study synapses and their structure–function relationship deep inside live brain tissue with high temporal and spatial resolution (12). In addition to imaging the morphology of fluorescently labeled neurons, two-photon microscopy allows for targeted stimulation of single synapses by photolysis of caged glutamate and other bioactive compounds (13) and measurements of molecular diffusion and enzymatic reactions using fluorescence recovery after photo-bleaching (FRAP) (14) and fluorescence lifetime imaging (FLIM) (15) in individual spines.

While being a powerful modality for imaging and stimulating neurons in living brain tissue, the spatial resolution of two-photon microscopy is limited by the diffraction of light to around 500 nm and, hence, falls short of resolving many important morphological details of neurons and glia cells. In particular spine necks, distal glial processes, and the shafts of axons have spatial dimensions of around 50–200 nm and, therefore, are not resolvable by two-photon microscopy (16).

For this reason, it has remained impossible to properly quantify the dynamics of these anatomical structures in live tissue, let alone to evaluate them relative to functional measurements. This is a major limitation for understanding the physiology of axons and spines, because their small size renders their functional properties particularly susceptible to minute morphological changes.

**Figure 1:**
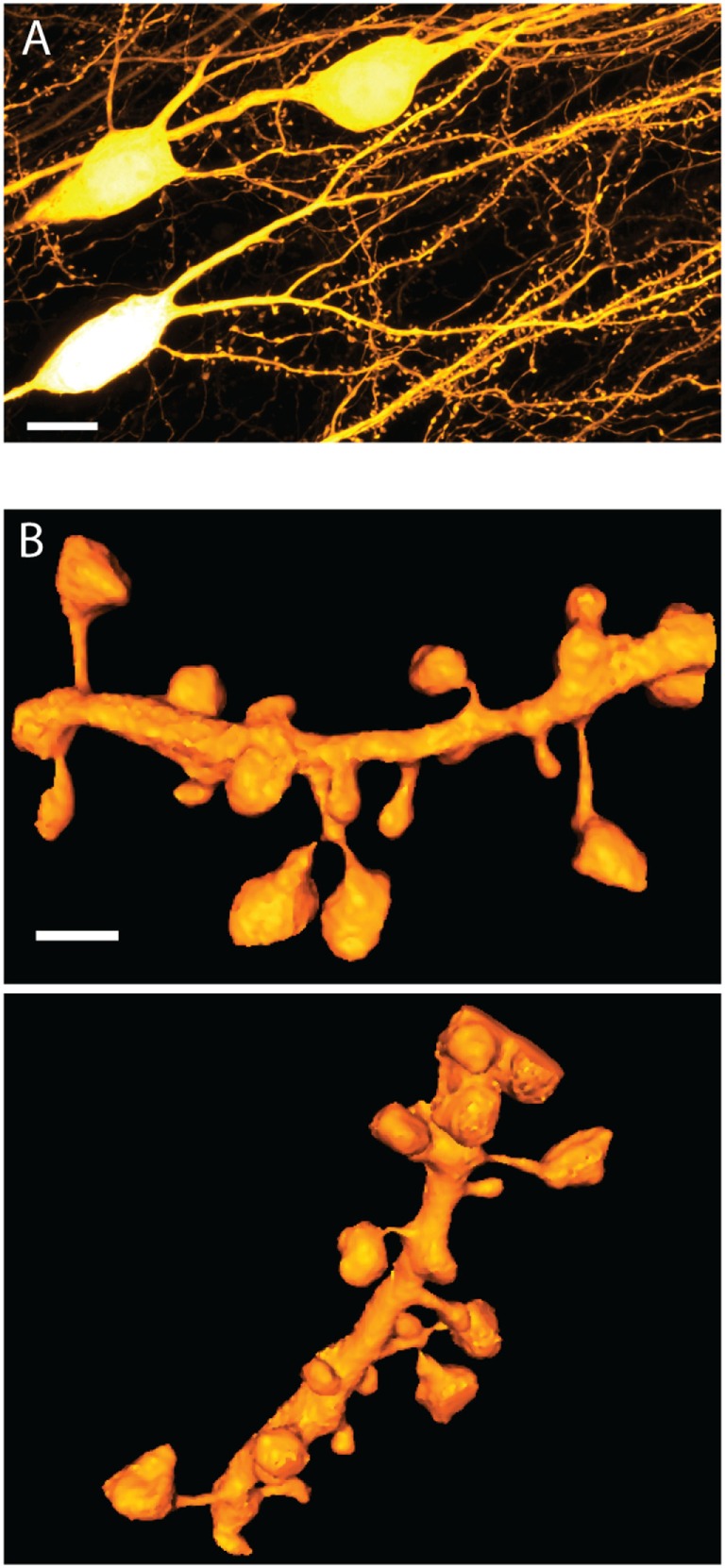
**Dendritic spine morphology**. **(A)** STED image of basal dendrites on live CA1 pyramidal cells in organotypic hippocampal slice prepared from Thy1-YFP transgenic animals. The image is a maximum intensity projection over 10 μm and is subjected to a 1-pixel median filter. Scale bar is 10 μm. **(B)** Two rotated views of a surface rendered 3D STED image of live spines on a dendritic segment in organotypic hippocampal slice as above. The rendering was prepared using an ImageJ 3D viewer plugin (17). The scale bar is 1 μm. Images acquired as in Ref. (18), with the addition of 3D STED (19).

Given their conspicuous morphology, typically featuring a bulbous spine head attached to the dendrite via an elongated neck, spines are bound to be immensely important for synapse physiology and neural plasticity (Figure 1). Indeed, activity-dependent remodeling of spines, such as changes in spine turnover and spine head size, has been a consistent finding across cell types and brain regions under a wide range of (patho-) physiological experimental conditions *in vitro* and *in vivo* (1).

Notably, a recent study showed that newly acquired motor skills can be disrupted by light-induced shrinkage of those spines that were potentiated during motor learning (20). Two other recent studies reported on spine changes in the hippocampus *in vivo* (21, 22), which is the brain area most closely associated with learning and memory formation. The reported rates of spine turnover were very different between these studies, which highlights the methodological challenge of visualizing spines over time in deeper brain regions.

The invention of fluorescence super-resolution STED microscopy (23, 24), which was recognized by the Nobel Prize in 2014, has substantially facilitated synapse imaging (25, 26). STED microscopy is not limited by the diffraction of light and allows visualization of even the finest details of synaptic structures and their dynamics in living brain tissue (27, 28). Initially restricted to just a few microns, the depth penetration of STED has been significantly extended to tens of micrometers tissue depth. This is achieved either by the use of two-photon excitation (29, 30), or glycerol objectives that match the refractive index of brain tissue better than oil objectives, and which are equipped with a correction collar to reduce the spherical aberrations from the residual refractive index mismatch (31).

In this review, we summarize our current understanding of the structure–function relationship of dendritic spines, and highlight current controversies and open questions. We discuss the potential impact of nanoscale spine structural plasticity on the electrical function of synapses, by relating recent live cell structural and functional data to earlier theoretical predictions.

## Spine Structure and Function

Spines stand out as unique neuro-anatomical specializations, and apart from their general head-and-neck design, no spine looks quite like any other (Figure 1). In fact, spine morphology is highly diverse, covering a broad distribution of shapes and sizes, which defies obvious categorization. Spine head volumes range from 0.01 to 1 μm^3^, while spine necks measure between 50 and 500 nm in diameter and are roughly up to 3 μm in length (32–34). Moreover, these morphological parameters show little correlation with each other.

Despite of this morphological continuum, spines are commonly grouped into a small number of distinct categories, such as stubby, mushroom, thin, and filopodial, based on their appearance (35). While this categorization scheme may be practical for analysis purposes, it is a gross over-simplification, where the categorization results depend strongly on image quality, which vary between studies. Moreover, image projection artifacts and limited spatial resolution mask short spine necks, which leads to the false identification of stubby spines (18).

There are consistent differences in the spectrum of their morphology across different dendritic locations and laminar positions, cell types, brain areas, animal age, and disease states (36), while the density of spines on dendrites is also highly variable; aspiny interneurons lack spines altogether, while cerebellar Purkinje cells carry more than 200,000 spines.

The ubiquity of dendritic spines across the phylogenetic tree points to a highly specialized and fundamental role; however, the rhyme and reason behind their remarkable structure and diversity remains enigmatic. Over the last decade, extensive experimental studies using EM or two-photon imaging combined with glutamate uncaging and electrophysiological approaches have established several ground rules for the relationship between their structure and function.

First and foremost, there is a broad consensus that the size of the spine head scales with the size of the PSD (32, 34), and the amplitude of the excitatory postsynaptic current (EPSC) (37, 38).

Accordingly, the induction of synaptic long-term potentiation (LTP) leads to spine head enlargement that scales with the potentiation of the EPSC (39–41). This structural effect primarily occurs in smaller spines (40), and is saturable as repeated rounds of induction lose their effectiveness, much like LTP (42).

While synaptic potentiation and spine enlargement occur within seconds after the induction protocol, the increase in PSD size develops more slowly over tens of minutes (43), indicating that multiple, kinetically distinct processes underlie the molecular and morphological remodeling of synapses.

In addition to modifications of existing spines, spines can grow *de novo* in response to a variety of triggers, including LTP-inducing electrical stimulation, two-photon glutamate uncaging, or altered sensory experience (44–47), leading to the formation of new functional synapses (48, 49).

Conversely, electrical induction of long-term depression (LTD) leads to shrinkage of the spine head and increased spine loss (45, 50), which can also be induced by glutamate uncaging (51, 52) and optogenetic stimulation (53).

Taken together, these studies support the view that during synaptic plasticity spine heads undergo size changes followed by remodeling of the PSD to accommodate a higher or lower number of receptors, depending on whether LTP or LTD is induced. According to this view, spines serve primarily as placeholders for the PSD and changes in postsynaptic strength are mediated by modulating the efficacy or number of synaptic receptors, e.g. Ref. (54).

Due to lack of spatial resolution, structural plasticity studies have traditionally been limited to reporting changes in spine numbers or spine head size, neglecting the spine neck, despite its potentially critical biophysical role as pointed out early on, as in Ref. (10, 55).

## Biochemical Compartmentalization in Spines

There is ample evidence that dendritic spines can spatially constrict the diffusion of second messenger molecules. Biochemical compartmentalization is thought to allow neurons to independently regulate each of their thousands of synapses, endowing the brain with an enormous information processing capacity.

The first experimental evidence for compartmentalized signaling came from calcium imaging studies showing that presynaptic stimulation can elicit calcium transients that are confined to single spines (56, 57). In addition, compartmentalized activation of a variety of signaling molecules, including second messengers and enzymes, has been demonstrated in spines after plasticity-inducing synaptic stimulation (58). Quantitative analyses of diffusion between spine and dendrite based on FRAP experiments demonstrate that diffusion rates vary widely between different spines, ranging from tens to hundreds of milliseconds for small fluorescent molecules (14, 59).

Interestingly, the diffusional coupling between spine and dendrite is reduced following repetitive stimulation of individual spines by two-photon glutamate uncaging (60), indicating that the degree of biochemical compartmentalization is subject to activity-dependent regulation.

These studies clearly established that spines form diffusionally isolated micro-compartments, even though the underlying biophysical mechanism remained unclear for a long time. While a correlation between FRAP time constant and spine neck length was observed (14, 59), additional intracellular factors, such as a meshwork of actin filaments or the spine apparatus (61, 62), are likely also to contribute to the diffusion barrier. Interestingly, micrometer-scale synaptic signaling domains exist even without spines in smooth dendrites of neocortical interneurons, suggesting that compartmentalization can be achieved in non-morphological ways (63).

**Figure 2:**
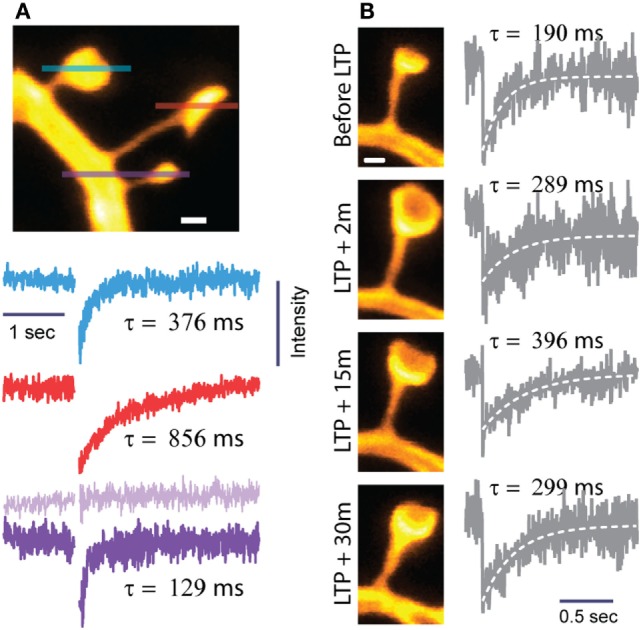
**Biochemical compartmentalization of dendritic spines**. Spine morphology defines the spine as a biochemical compartment. **(A)** Neighboring spines often differ widely in shape and size and, hence, compartmentalize diffusive signals in a very different way. The lines through the spines show where FRAP was recorded by line scanning. The FRAP traces and their diffusional time constants (τ) correspond to the individual spines according to the color code and sequence (top to bottom). Spines with thin, long necks and large heads experience slower diffusional recovery and, hence, higher τ values. Modified from Ref. (18). **(B)** Induction of LTP by glutamate uncaging triggers structural changes in spine heads and necks, which have opposite effects on compartmentalization, so that τ changes less than predicted by looking at either neck or head dynamics alone [modified from Ref. (18)]. Scale bars are 0.5 μm.

Combining FRAP experiments with super-resolution imaging allows for direct comparisons of molecular diffusion and nanoscale morphology in identified spines (Figure 2). Through this approach, we recently found that more than half of the measured variation in FRAP time constants across spines can be accounted for by spine morphology (18). While it is clear that the diffusional properties of spines are strongly shaped by spine morphology, there is still considerable variation that may be explained by other factors, such as organelles or cytoskeletal structures in the spine head and neck.

By first approximation, the diffusional FRAP time constant τ depends on spine morphology as follows (14, 64):

(1) $$\tau =\frac{V\times L}{A\times D}$$

where *V* is the volume of the spine head, *L* the length, *A* the cross-sectional area of the spine neck, and *D* the diffusion coefficient of the fluorophore.

This simple formula shows that changes in τ can be realized in different ways: τ will increase if the spine head enlarges or if the neck becomes longer or thinner. Parallel changes in head-and-neck morphology may be additive or cancel each other out regarding their effects on overall compartmentalization. For instance, τ will stay more or less constant if the spine head becomes larger and the spine neck widens at the same time.

These distinctions are pertinent given that the induction of LTP not only enlarges spine heads (40) but also leads to shorter and wider spine necks, so that τ changes less than what would be predicted if only one parameter were to change (Figure 2) (18).

While τ remains largely unaltered after LTP, the biophysical environment of the synapse and the compartmental properties of the spine are certainly affected, as the increase in spine head size will effectively lower the concentration of molecules released into the enlarged spine volume, and more permissive spine necks will facilitate the exchange of material (molecules, vesicles, organelles) between the spine head and parent dendrite.

Finally, it is worth mentioning that spine morphology is likely to influence other diffusion-dependent processes, including the spread of chloride in dendritic shafts, which impacts short-term plasticity of GABAA receptor signaling and inhibitory drive (65) and the mobility and trafficking of synaptic receptors and synaptic scaffold proteins within nano-domains that have been recently reported (66, 67).

## Electrical Compartmentalization of Dendritic Spines

In contrast to biochemical compartmentalization, the case for electrical compartmentalization remains highly controversial, primarily due to technical limitations in measuring electrical signals directly in the spine, which forces experimenters to infer them by indirect means.

**Figure 3:**
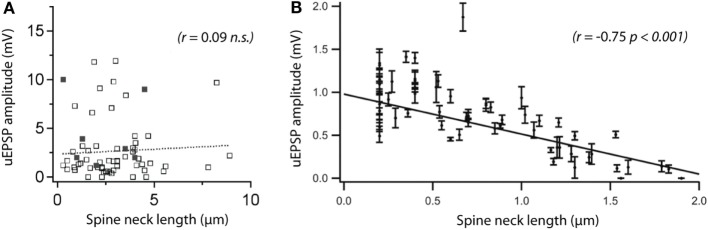
**Are spines capable of compartmentalizing electrical signals?** There is no consensus on the role of the spine neck in electrical signaling, and conflicting results have been reported. **(A)** A recent two-photon microscopy study comparing spine morphology with uncaging (u)EPSP amplitude did not see a correlation between somatic uEPSPs and neck length. The solid dots represent spontaneous synaptic activity (evaluated by calcium imaging). Reprinted from Bywalez et al. (74), with permission from Elsevier. **(B)** Using a similar experimental approach, a previous study reported a strong correlation between the same parameters. The discrepancy between the two studies adds to an ongoing controversy about the importance of the spine neck in electrical compartmentalization of synapses. Modified with permission from Ref. (69) Copyright (2006) National Academy of Sciences, USA.

Several early studies based on cable theory (55, 68) and FRAP experiments (14) indicated that spines cannot modify synaptic signals appreciably. Subsequent experimental work based on Ca^2+^ imaging, two-photon glutamate uncaging, electrophysiology, and mathematical modeling has pointed to the contrary, indicating that spines are sufficiently electrically isolated to impact synaptic potentials and their dendritic integration (69–71). More recently, the pendulum has swung back, with studies based on voltage-sensitive dye imaging (72) and super-resolution STED microscopy (73), arguing that the spine neck has no effect on synaptic signals in the dendrite or soma. The lack of consensus effectively leaves open the basic question of the impact of spine morphology on the electrical signaling of synapses (Figure 3).

### Modeling Voltage Transfer in Dendritic Spines

**Figure 4:**
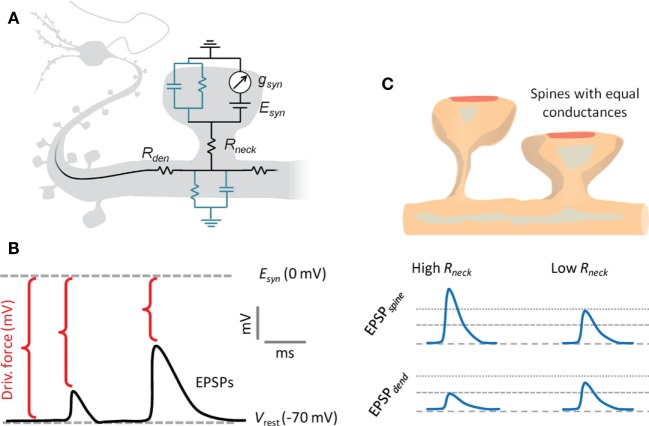
**Electrical compartmentalization of dendritic spines**. **(A)** In the spine electrical circuit diagram, a variable current enters through the synaptic receptors, scaling with their conductance, *g*_syn_, and with the electrical driving force, which is the difference between resting membrane potential and the reversal potential of the conductance, *E*_syn_. The membrane resistance is so high that current will not escape, and it will instead pass first the neck resistance, *R*_neck_, and then the dendritic input resistance, *R*_dendrite_, on the way to the soma. The EPSP that the synaptic current generates along the way is defined by Ohm’s law and follows voltage divider law. **(B)** As the synaptic current scales with driving force, the depolarizing EPSPs produced by the current will have a self-dampening effect as they approach the glutamate receptor reversal potential, *E*_syn_. **(C)** A thin and long spine neck will have a high *R*_neck_, which will locally boost the EPSP in the spine head. This in turn causes a loss of driving force, so that less current will flow over the synaptic conductance. While the EPSP in the spine head sees both the boosting and the loss of driving force, the corresponding EPSP in the dendrite only experiences the loss of driving force. Conversely, a spine with a low *R*_neck_ will see less boosting of the spine head EPSP and less current attenuation, so the spine and dendritic EPSPs are more similar. Beyond the illustrated passive effects of morphology, the boosted spine head EPSP may locally recruit voltage-gated conductances on the spine, which may in turn increase or decrease the synaptic current.

To gain insights into how spine morphology may influence synaptic signaling, we will consider an equivalent electrical circuit, which models the electrical phenomena in the postsynaptic neuron at steady state (Figure 4) (55). The model does not take into consideration the membrane capacitance and active conductances other than the ligand-gated synaptic conductance. Therefore, the synaptic current is modeled to flow without capacitive losses or active amplification from the spine head to the dendrite.

We point out already here that the spine neck will simultaneously have differential effects on the voltage in the spine head and the dendrite, and that the effects in the spine head are more pronounced in absolute voltages (Figure 4). However, only the effects manifested on the dendritic side will matter for dendritic integration and action potential firing.

When an excitatory synapse is stimulated, glutamate receptors (primarily of the AMPA type, but also NMDA) open, causing a net inward ionic current. The synaptic current (*I*_syn_) scales with the synaptic conductance (*g*_syn_) and driving force:

(2) $$I_{\text{syn}}=g_{\text{syn}}\times \left(V_{\text{spine}}-E_{\text{syn}}\right)$$

where *V*_spine_ is the voltage in the spine head, *E*_syn_ is the reversal potential of the synaptic conductance (around 0 mV for glutamate receptors), and the term *V*_spine_ − *E*_syn_ denotes the driving force (around 70 mV).

**Figure 5:**
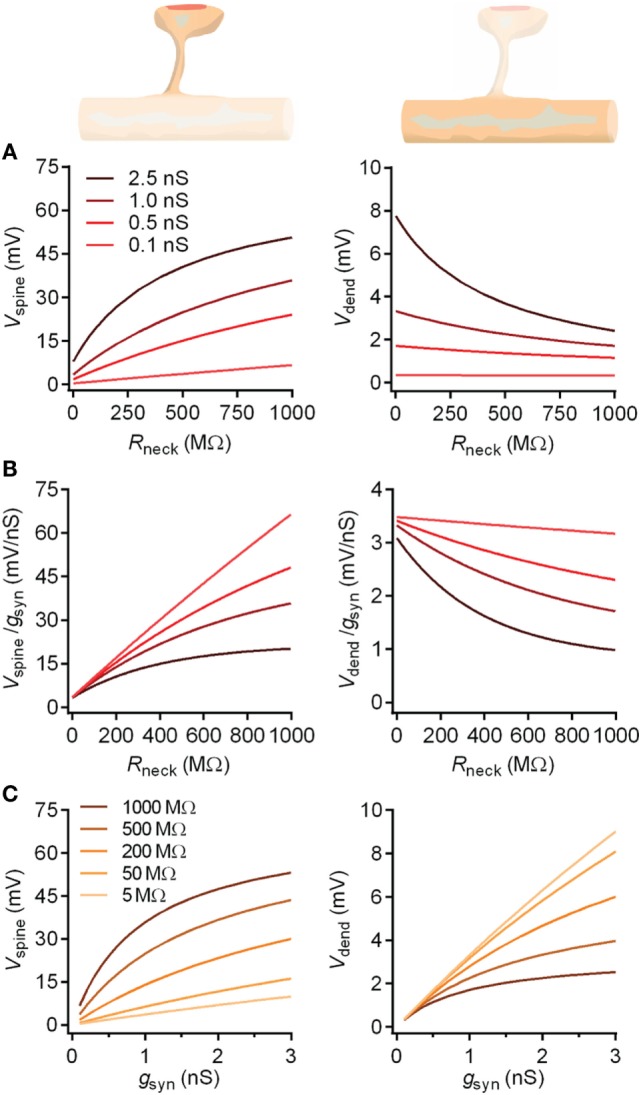
**The impact of the spine neck resistance on EPSPs in spine heads and dendrites**. The spine neck resistance has opposite effects on EPSPs in the spine head and in the dendrite. **(A)** For a given conductance, the neck will boost the spine head voltage, which in turn will reduce driving force and saturate the boosting effect. By contrast, in the dendrite, the neck no longer boosts the voltage, and only the reduced driving force is manifested as a decreasing voltage with increasing *R*_neck_. Both effects are more pronounced for synapses with higher conductances, as these produce higher voltages and stronger reductions in driving force. **(B)** This simultaneous boosting and saturation effect of the neck is manifested as a reduced voltage per conductance (synaptic gain) as a function of *R*_neck_, which is more pronounced for stronger synapses. Again, the dendrite sees only the saturation effect, while the spine voltage is also boosted. **(C)** Conversely, for a fixed *R*_neck_ value, increasing the synaptic conductance will boost both the spine and dendritic voltages, with an accordingly stronger saturation effect if *R*_neck_ is higher. From the different *R*_neck_ values plotted, it is evident that while *R*_neck_ boosts the spine voltage, it simultaneously attenuates the dendritic voltage.

The amplitude of the excitatory postsynaptic potential (EPSP) in the spine head (*ΔV*_spine_ = *V*_spine_ − *V*_rest_) can be described by the following equation:

(3) $$\Delta V_{\text{spine}}=\frac{g_{\text{syn}}\times \left(R_{\text{neck}}+R_{\text{dendrite}}\right)\times \left(E_{\text{syn}}-V_{\text{rest}}\right)}{1+g_{\text{syn}}\times \left(R_{\text{neck}}+R_{\text{dendrite}}\right)}$$

where *V*_rest_ is the resting membrane potential (typically around −70 mV), *R*_neck_ the electrical resistance of the spine neck, and *R*_dendrite_ the dendritic input resistance at the location of the spine (Figure 5).

It is instructive to consider the two limiting cases of Eq. 3, where both *g*_syn_ and *R*_neck_ are either very small or very large, respectively. More precisely, if *g*_syn_ × (*R*_neck_ + *R*_dendrite_) ≪ 1, the expression for Δ*V*_spine_ simplifies to:

(4) $$\Delta V_{\text{spine}}=g_{\text{syn}}\times \left(R_{\text{neck}}+R_{\text{dendrite}}\right)\times \left(E_{\text{syn}}-V_{\text{rest}}\right)$$

In this regime, the deflection in spine head voltage is a small fraction of *V*_rest_ and depends linearly on *g*_syn_, and the sum of the electrical resistances, *R*_neck_ and *R*_dendrite_. The spine effectively acts as a current source, meaning that the synaptic current is independent of the downstream electrical resistance.

In the opposite limiting case, when the synaptic conductance and synapse input resistance are very large, i.e., if *g*_syn_ × (*R*_neck_ + *R*_dendrite_) ≫ 1, the expression is reduced to:

(5) $$\Delta V_{\text{spine}}=\left(E_{\text{syn}}-V_{\text{rest}}\right)\approx 70\text{ mV}$$

In this regime, the spine head voltage approaches the reversal potential of the synaptic conductance (0 mV) and, thus, becomes independent of any other parameter, including *R*_neck_. In contrast to the former case, the spine now acts like a constant voltage source, effectively clamping the voltage to 0 mV in the spine head.

After entering the spine head, the synaptic current passes through the spine neck into the dendrite, spreading mostly to the somatic region, from where it exits the cell. Along the way, the current causes local changes in membrane voltage, leading to the EPSP in the soma, which can be measured electrophysiologically.

From the spine head to the dendrite, the voltage drops according to the voltage divider law, yielding a voltage signal (Δ*V*_dendrite_) at the adjacent dendritic location:

(6) $$\Delta V_{\text{dendrite}}=\frac{R_{\text{dendrite}}}{R_{\text{neck}}+R_{\text{dendrite}}}\Delta V_{\text{spine}}$$

which, given Eq. 3, can be expressed as (Figure 5):

(7) $$\Delta V_{\text{dendrite}}=\frac{g_{\text{syn}}\times R_{\text{dendrite}}\times \left(E_{\text{syn}}-V_{\text{rest}}\right)}{1+g_{\text{syn}}\times \left(R_{\text{neck}}+R_{\text{dendrite}}\right)}$$

Also here, it is interesting to consider the two limiting cases for Eq. 7. In the case of *g*_syn_ × (*R*_neck_ + *R*_dendrite_) ≪ 1, the voltage deflection in the dendrite becomes:

(8) $$\Delta V_{\text{dendrite}}=g_{\text{syn}}\times R_{\text{dendrite}}\times \left(E_{\text{syn}}-V_{\text{rest}}\right)$$

which is similar to Eq. 4, except now the voltage only depends on *R*_dendrite_. An important implication is that any changes in *R*_neck_ will be inconsequential for the dendritic voltage as long as the limiting case applies.

By contrast, in the case of *g*_syn_ × (*R*_neck_ + *R*_dendrite_) ≫ 1, the dendritic EPSP is as follows:

(9) $$\Delta V_{\text{dendrite}}=\frac{R_{\text{dendrite}}}{R_{\text{neck}}+R_{\text{dendrite}}}\times \left(E_{\text{syn}}-V_{\text{rest}}\right)$$

which means that *R*_neck_ and *R*_dendrite_ determine the EPSP amplitude in the dendrite and soma, and that changes in spine neck dimensions can directly affect this.

### Electrical Resistance of the Spine Neck

The key parameters to consider in this discussion are *g*_syn_, *R*_neck_, and *R*_dendrite_, because they determine the amplitude of the current entering the synapse and the resultant voltages in the spine head and dendrite. While *g*_syn_ and *R*_dendrite_ can be reasonably well determined by patch-clamp recordings, measuring *R*_neck_ is much more difficult, because of the inaccessibility of the spine head for electrophysiological recordings. However, this important biophysical parameter can be estimated in several indirect ways, all of which have specific advantages and caveats.

(1) The spine neck can be modeled as a passive ohmic resistor, which is defined by its cross-sectional area (*A*), length (*L*), and cytoplasmic electrical resistivity (ρ). *R*_neck_ can be then be calculated by the formula (9, 10):
  (10) $$R_{\text{neck}}=\frac{\rho \times L}{A}$$
  Given sufficiently resolved images of dendritic spines, this morphology-based estimate is straightforward. However, it ignores the intracellular constituents of the spine neck, such as the spine apparatus or other organelles, which are likely to affect the electrical resistance of the spine neck.
  Based on spine morphology obtained from EM images, and assuming a value of 100 Ωcm for ρ, spine neck resistances were estimated to range between 1 and 400 MΩ for CA1 pyramidal neurons (32). We recently reported a similar range for live spines, between 2 and 600 MΩ, based on STED microscopy in brain slices (18).
(2) *R*_neck_ can be also estimated by FRAP experiments (Figure 2). After bleaching a substantial fraction of small diffusible fluorophores inside the spine head (which is equivalent to a concentration jump), the time constant of fluorescence recovery (τ) is related to *R*_neck_, ρ, the spine head volume *V*, and the diffusion coefficient *D* according to the formula (14):
  (11) $$R_{\text{neck}}=\frac{\tau \times \rho \times D}{V}$$
  This method has the advantage that it is sensitive to contributions from intracellular factors, and does not require any knowledge of spine neck morphology, only the volume of the spine head that is easier to estimate.
  Using this strategy, more variable ranges have been reported for *R*_neck_, between 4 and 150 MΩ (14), up to 1 GΩ (60), and between 5 MΩ and 1.2 GΩ (18).
(3) *R*_neck_ has been estimated based on a combination of calcium imaging and modeling, where voltage-dependent calcium channels are used as a sensor of the voltage deflection in the spine head. However, the calcium fluorescence signal depends on the voltage in a highly non-linear way, which makes quantitative measurements challenging.
  Based on this method the reported range is between 400 and 800 MΩ (71) and up to 1.2 GΩ (70). These values are generally higher and show less variation than the estimates based on morphology and FRAP. However, the discrepancies might reflect measurement biases, where spines with high neck resistances produce larger and, hence, more detectable calcium transients than spines with lower neck resistances.
(4) Finally, voltage imaging in dendritic spines is emerging as a new method, which may, in principle, provide a direct measure of *R*_neck_. While holding great promise, optical detection of sub-threshold voltage deflections in spatial micro-compartments still poses considerable challenges concerning signal sensitivity, accuracy, and calibration.

A recent study based on voltage-sensitive dye imaging in spines on thin basal dendrites of cortical pyramidal neurons provided an estimate of *R*_neck_ around 27 MΩ (72), contrasting sharply with previous higher estimates from calcium imaging (70, 71), although still falling within the low-end range of the FRAP and morphological estimates (14, 18, 32).

It is obvious that there is substantial disagreement in the literature on the mean value and variability of *R*_neck_, and it remains unclear to what extent these discrepancies reflect physiological (brain area, cell type, etc.) or methodological (accuracy, experimental preparation, temperature, etc.) differences.

However, given the available evidence, it seems likely that *R*_neck_ varies widely, ranging from a few mega ohms to at least several hundred mega ohms. This variability implies that electrical compartmentalization of spines is also highly variable. Assuming a value of 50 MΩ for *R*_dendrite_, the spine head voltage may be similar or more than ten times larger than the dendritic EPSP, depending on the value of *R*_neck_.

## Regulation of Synaptic Strength through Structural Plasticity

It is a long-standing question whether spine structural plasticity represents a mechanism to tune synaptic strength. While the basic idea was conceived decades ago (8, 75, 76), it has laid largely dormant after being dismissed on theoretical grounds (9) and given the technical difficulties to explore it experimentally.

While it is clear that spine head enlargement or shrinkage is associated with functional changes, structural plasticity has essentially been viewed as a mere space issue: changes in spine head size reflect a dynamic capacity to accommodate a higher or lower number of synaptic receptors or scaffolding proteins. Hence, changes in synaptic strength are usually attributed to mechanisms that converge on modifying the conductance level of the synapse, through changes in presynaptic release probability, the clearance of glutamate from the synaptic cleft, or the number and biophysical properties of synaptic receptors. From this conductance-centric perspective, structural plasticity plays a permissive role for functional plasticity, but in and of themselves structural changes do not have direct effects on synaptic transmission.

More than 30 years ago, pioneering work based on EM provided the first indirect evidence for spine neck plasticity (7). But being limited to fixed preparations EM could not provide a smoking gun, and this work was ignored until 20 years later, when two-photon microscopy was able to provide time-lapse evidence for neck changes in live spines.

However, the scarce published results have been conflicting; on the one hand, neuronal activity was shown to slow down diffusion across the spine neck (60) and, on the other hand, it was shown to drive spine neck shortening (77, 78), which should rather facilitate diffusion.

Using super-resolution STED microscopy in combination with two-photon glutamate uncaging and patch-clamp electrophysiology, we obtained direct evidence that spine necks become shorter and wider after the induction of LTP, while the spine head is enlarged and the synaptic conductance increased (Figure 3) (18). Based on the morphological estimate of spine neck resistance (Eq. 10), these structural changes amount to a major reduction (on average by 50%) in *R*_neck_.

In light of our discussions above, if the synapse operates in the current source regime, a change in *R*_neck_ will only affect the voltage in the spine head, whereas if it acts as a voltage source, it will only influence the dendritic EPSP. In reality, most synapses are likely to occupy a middle ground between these two extreme regimes, so that spine neck plasticity might simultaneously influence synaptic signals in the spine and dendrite.

Hence, a reduction in *R*_neck_ is likely to have at the same time differential effects on the EPSP on either side of the spine neck, lowering it in the spine head, while elevating it in the dendrite. Conversely, an increase in *R*_neck_ will boost the voltage in the spine head and lower it in the dendrite (Figure 5). The actual magnitude of the effects will depend on the relative sizes of the parameters *g*_syn_, *R*_neck_, and *R*_dendrite_, according to the formulas above (Eqs. 3 and 7).

Counterintuitive at first sight, the local drop in spine head EPSP is actually facilitating LTP, because it reduces the negative feedback on the synaptic current resulting from a loss of driving force, which occurs as the spine head voltage approaches the synaptic reversal potential and effectively saturates. This negative feedback between spine EPSPs and driving force may under normal conditions be quite pronounced in spines with long and thin necks, which have high *R*_neck_ values. Thus, reducing *R*_neck_ may be a physiological mechanism during LTP, whereby the investment of increasing synaptic receptor numbers (i.e., synaptic conductance) is protected by counteracting voltage saturation in the spine head.

Beyond the immediate effects on EPSPs, large changes in *R*_neck_ might effectively shift the operating regime of the synapse, acting more like a voltage or current source. Such a major “parametric” change would modify the voltage transformation of the synapse and may, thus, affect dendritic integration and the computational performance of the neuron.

### Secondary Effects on Active Conductances by Structural Plasticity

By influencing the spine head EPSP, changes in *R*_neck_ might strongly affect the activation of voltage-gated ion channels in the spine head, such as voltage-sensitive calcium and sodium channels, which in turn shape the EPSP (74, 79). Likewise, the voltage-dependent block of the NMDA receptor by extracellular magnesium will be directly affected by changes in the spine head EPSP.

At present, it is hard to make quantitative predictions on how electrical signaling at the synapse will be affected by these highly non-linear and dynamic interactions. Modeling can provide some intuitive insights; however, the results of numerical simulations will depend steeply on the model parameters for the active and passive properties of the synapse, many of which are still poorly known.

## Outlook

Ever since the discovery of dendritic spines by Ramon y Cajal, generations of neuroscientists have peeled away layers of their secrets. Yet, a comprehensive understanding of their structure–function relationship remains elusive, and continues to pose one of the great challenges in neuroscience.

The development of powerful optical microscopy techniques, such as super-resolution microscopy, two-photon glutamate uncaging, and voltage-sensitive dye imaging, is making it increasingly possible to measure key biophysical parameters with sufficient sensitivity and spatial and temporal resolution under a variety of physiologically relevant experimental conditions. Together with computer simulations, these new techniques will transform our understanding of the role of spines for synaptic function, neural computation, and ultimately behavior.

## Author Contributions

All authors listed have made substantial, direct, and intellectual contribution to the work and approved it for publication.

## Conflict of Interest Statement

The authors declare that the research was conducted in the absence of any commercial or financial relationships that could be construed as a potential conflict of interest.
